# Cell Polarity and Patterning by PIN Trafficking through Early Endosomal Compartments in *Arabidopsis thaliana*

**DOI:** 10.1371/journal.pgen.1003540

**Published:** 2013-05-30

**Authors:** Hirokazu Tanaka, Saeko Kitakura, Hana Rakusová, Tomohiro Uemura, Mugurel I. Feraru, Riet De Rycke, Stéphanie Robert, Tatsuo Kakimoto, Jiří Friml

**Affiliations:** 1 Department of Plant Systems Biology, Flanders Institute for Biotechnology (VIB), Gent, Belgium; 2 Department of Plant Biotechnology and Genetics, Ghent University, Gent, Belgium; 3 Department of Biological Sciences, Graduate School of Science, Osaka University, Osaka, Japan; 4 Institute of Science and Technology Austria (IST Austria), Klosterneuburg, Austria; 5 Department of Biological Sciences, Graduate School of Science, University of Tokyo, Tokyo, Japan; University of Natural Resources and Life Sciences, Austria

## Abstract

PIN-FORMED (PIN) proteins localize asymmetrically at the plasma membrane and mediate intercellular polar transport of the plant hormone auxin that is crucial for a multitude of developmental processes in plants. PIN localization is under extensive control by environmental or developmental cues, but mechanisms regulating PIN localization are not fully understood. Here we show that early endosomal components ARF GEF BEN1 and newly identified Sec1/Munc18 family protein BEN2 are involved in distinct steps of early endosomal trafficking. BEN1 and BEN2 are collectively required for polar PIN localization, for their dynamic repolarization, and consequently for auxin activity gradient formation and auxin-related developmental processes including embryonic patterning, organogenesis, and vasculature venation patterning. These results show that early endosomal trafficking is crucial for cell polarity and auxin-dependent regulation of plant architecture.

## Author Summary

Auxin is a unique plant hormone, which is actively and directionally transported in plant tissues. Transported auxin locally accumulates in the plant body and triggers a multitude of responses, including organ formation and patterning. Therefore, regulation of the directional auxin transport is very important in multiple aspects of plant development. The PIN-FORMED (PIN) family of auxin transporters is known to localize at specific sides of cells and export auxin from the cells, enabling the directional transport of auxin in the tissues. PIN proteins are rapidly shuttling between the plasma membrane and intracellular compartments, potentially allowing dynamic changes of the asymmetric localization according to developmental and environmental cues. Here, we discovered that a mutation in the Sec1/Munc18 family protein VPS45 abolishes its own early endosomal localization and compromises intracellular trafficking of PIN proteins. By genetic and pharmacological inhibition of early endosomal trafficking, we also revealed that another early endosomal protein, ARF GEF BEN1, is involved in early endosomal trafficking at a distinct step. Furthermore, we showed that these components play crucial roles in polar localization and dynamic repolarization of PIN proteins, which underpin various developmental processes. These findings highlight the indispensable roles of early endosomal components in regulating PIN polarity and plant architecture.

## Introduction

Plant hormone auxin locally accumulates in plant tissues and regulates multiple processes of plant growth and development [1], [2]. Directional intercellular transport of auxin underlies most of known auxin-dependent control of development, including embryogenesis, root and shoot organogenesis, vascular tissue formation and asymmetric phototropic and gravitropic growths [3]. This polar auxin transport is achieved by collective actions of auxin efflux and influx transporters [4]–[6]. PIN-FORMED (PIN) family proteins asymmetrically localize at the plasma membrane (PM) in different plant tissues [7] and exhibit auxin efflux activities [8]. The polar localization of PIN proteins, together with their molecular role as auxin efflux facilitators, correlates well with known direction of polar auxin transport in different plant tissues. Furthermore, manipulation of polar PIN localization causes changes in auxin distribution and altered developmental and/or growth responses [7], [9]. Supported by these lines of evidence, it is widely accepted that polar localization of PIN proteins is essential in regulating auxin distribution in plant tissues.

Detailed observations of PIN family proteins have revealed that their polar localization changes dynamically during plant development [10]–[12] including responses to environmental cues [13]–[15]. PIN proteins are rapidly and constitutively shuttling between the PM and endosomes, providing a potential mechanism for their dynamic relocation [16], [17]. Fungal toxin brefeldin A (BFA) is known to inhibit vesicle transport that involves GDP-GTP exchange factors for small G proteins of ARF class (ARF GEFs). In *Arabidopsis thaliana* root, recycling of PIN1 protein preferentially to the basal side of cells requires a GBF-type ARF GEF, GNOM, which is highly sensitive to BFA [18]. As such, treatment with BFA of *Arabidopsis* roots results in intracellular accumulation of PIN1 proteins in agglomerated endomembrane compartments called ‘BFA compartments’[16].

By using BFA as a tool to visualize early endocytic trafficking defects, we have identified *bfa-visualized endocytic trafficking defective* (*ben*) mutants, which accumulate less PIN1-GFP proteins in BFA compartments [19]. *BEN1* encodes a putative ARF GEF, which belongs to BIG class of ARF GEF subfamily and localizes to early endosomes [19]. However, information on the molecular components involved in endocytic trafficking remains scarce. It has also been elusive to what extent the early endosomal trafficking events are important for polar localization of proteins and thus to polarized development.

To gain better understanding of early endosomal trafficking in plants, we identified additional regulators of this process, manipulated it by genetic and pharmacological means and revealed its impact on cell polarity and development.

## Results

### *BEN1* and *BEN2* are involved in different steps of early endosomal trafficking

**Figure 1:**
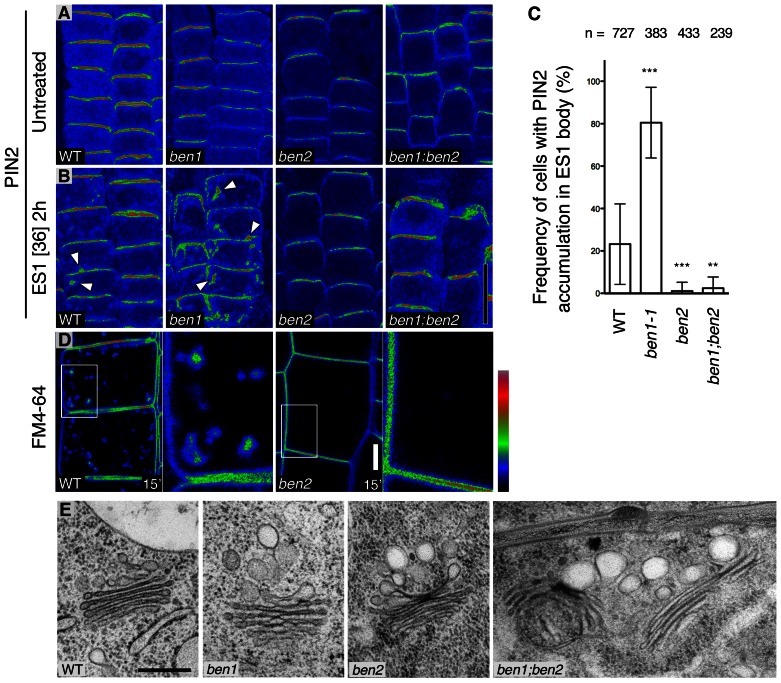
BEN1 and BEN2 are involved in distinct steps of early endosomal trafficking. (A,B) PIN2 immunofluorescence signals in root epidermal cells without drug treatment (A) and after ES1 treatment (36 µM, 2 h) (B). Arrowheads indicate intracellular agglomerations of PIN2 signals. (C) Quantitative analyses of intracellular accumulation of PIN2 in ES1 treated root epidermal cells. Asterisks indicate significant difference from wild type control (**: P<0.01; ***: P<0.0001 by t-test). Error bars indicate standard deviation among individual roots. N: number of cells examined. (D) Uptake of endocytic tracer FM4-64 in wild type (left) and *ben2* (right) root epidermal cells, 15 minutes after the onset of FM4-64 labeling. Magnified views of the boxed regions are indicated. Signal intensity is represented by the color code as indicated. (E) Ultrastructure of membranes associated with the Golgi apparatus in root epidermal cells of wild type and mutants. Scale bars: 20 µm in (B) for (A,B); 5 µm for (D); 300 nm for (E).

To dissect the early endosomal trafficking pathway in *Arabidopsis* root epidermal cells, we examined effects of a chemical inhibitor Endosidin1 (ES1), which affect actin dynamics and interfere with trafficking of endocytic cargoes at the *trans*-Golgi-network/early endosome (TGN/EE) [20], [21]. As shown previously [20], ES1 causes accumulation of PIN2 in agglomerated intracellular compartment (ES1 body) in wild type root epidermal cells (Figure 1A, 1B). Remarkably, accumulation of PIN2 protein in ES1 body was more pronounced in *ben1* mutants than in wild type, indicating that *ben1* mutation and ES1 treatment synergistically inhibited trafficking at the TGN/EE (Figure 1B, 1C).

Similar examination of *ben2* mutant, which exhibits reduced agglomeration of PM proteins upon BFA treatment [19] (Figure S1A), revealed a less pronounced intracellular accumulation of PIN2 upon ES1 treatment (Figure 1B, 1C). The distinct responses to ES1 prompted us to determine the genetic relationship between *ben1* and *ben2*. After incubation with ES1 under the same condition, *ben1; ben2* double mutant cells did not show strong intracellular accumulation of PIN2 as compared with *ben1* mutant (Figure 1B, 1C), indicating that *ben2* mutation is epistatic in terms of responses to ES1.

Next, we tested if *ben2* mutation affects endocytic trafficking by using a lipophilic styryl dye FM4-64, which is commonly used as endocytic tracer. As shown in Figure 1D, accumulation of endocytosed FM4-64 in endosomal compartments was also decreased in *ben2*. At later time point, however, FM4-64 stained vacuolar membrane in wild type as well as *ben2* mutant (Figure S1B), implying that endocytic transport is operational to some extent in *ben2* mutant. Therefore, it seems that BEN2 promotes transport of endocytosed cargo proteins to ES1-sensitive compartment (i.e. TGN/EE), whereas BEN1 functions in an ES1-insensitive pathway promoting transport of cargo-containing vesicles from the TGN/EE. As these observations indicated that BEN1 and BEN2 are functionally required for trafficking at the TGN/EE, we examined morphology of this compartment. Ultrastructural analysis revealed that enlarged vesicle-like structures often associated with the Golgi apparatus in *ben1* and *ben2* mutant cells (Figure 1E, Figure S1C). Additive features of both mutants were observed in the double mutant cells (Figure 1E, Figure S1C). Consistently, *ben1* and *ben2* mutations had a synergistic effect on seedling growth (Figure S1D, S1E). Taken together, these observations suggest the distinct roles of BEN1 and BEN2 in intracellular trafficking processes at the TGN/EE.

### *BEN2* encodes a Sec1/Munc18 family protein AtVPS45

**Figure 2:**
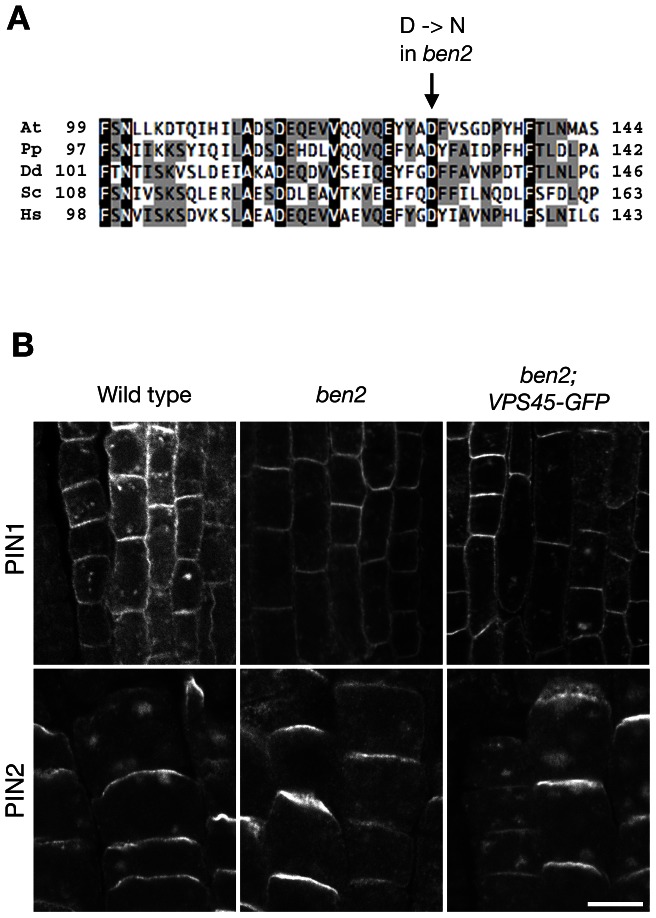
*BEN2* encodes a SM protein AtVPS45. (A) Alignment of VPS45 protein sequences from various organisms. At: *Arabidopsis thaliana*, (At1g77140); Pp: *Physcomitrella patens* (XP_001758633); Dd: *Dictyostelium discoideum* (XP_635835); Sc: *Saccharomyces cerevisiae* (NP_011420); Hs: *Homo sapiens* (NP_009190). (B) Complementation by VPS45-GFP of *ben2* mutant phenotypes. After BFA treatment (25 µM for 2 h), PIN1 accumulated in intracellular compartments in wild type root vascular tissue. Whereas intracellular accumulation of PIN1 was less pronounced in *ben2* mutant, clear accumulation of PIN1 was detected in *ben2* mutant harboring VPS45-GFP. Similarly, PIN2 accumulation in root epidermal cells upon BFA treatment was recovered in *ben2*; *VPS45-GFP*. Scale bar: 10 µm.

To clone the *BEN2* gene, we narrowed down the chromosomal region carrying *ben2* mutation to a 140-kb region by fine mapping (Figure S2A). Subsequently, we took a candidate approach and found a mutation in At1g77140 gene, encoding a Sec1/Munc18 (SM) family protein VACUOLAR PROTEIN SORTING 45 (AtVPS45) [22]. The mutation in *ben2* was predicted to cause amino-acid substitution of aspartic acid at position 129, which is well conserved in VPS45 of eukaryotic organisms, to asparagine (Figure 2A, Figure S2B). In *ben2* mutant transformed with *VPS45-GFP* transgene, BFA responses as well as growth defects were restored (Figure 2B, Figure S2C), confirming that the mutation in *VPS45* is responsible for *ben2* phenotypes.

### BEN2/VPS45 resides in early endocytic route

**Figure 3:**
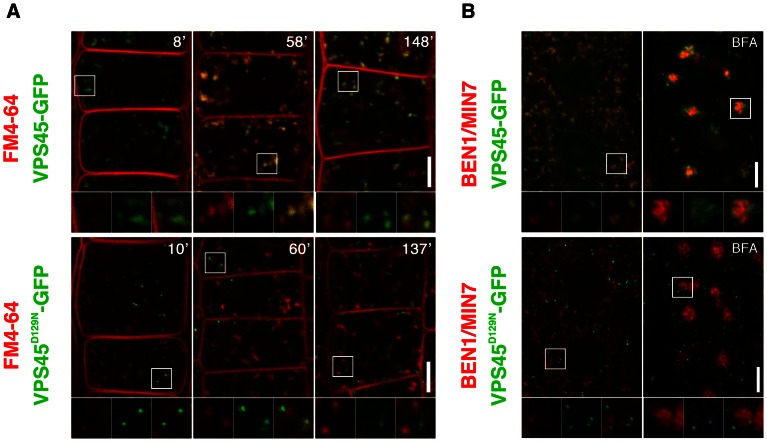
Double labeling experiments reveal early endosomal localization of VPS45 in root epidermal cells. (A) Wild type VPS45-GFP (green) colocalized with FM4-64 (red) within 10 minutes after the onset of staining. Additional endocytic compartments were labeled by prolonged incubation (upper panels). Mutated VPS45-GFP carrying *ben2* mutant sequence (VPS45D129N-GFP, green) did not colocalize with FM4-64 under the same conditions (lower panels). (B) Immunostaining of BEN1 (red) and VPS45-GFP (green). Whereas wild type version of VPS45-GFP partially colocalized with BEN1 (upper left panel), *ben2* mutation abolished the colocalization (lower left panel). BEN1 and VPS45-GFP responded differently to BFA (upper right panel). Whereas BEN1 accumulates to the center of the BFA compartment in BFA-treated cells, majority of VPS45-GFP localized to the periphery of the BFA compartment. *ben2* mutation caused mislocalization of the VPS45-GFP protein (green), although it did not affect the agglomeration of BEN1 signal (lower right panel). Magnified views of the regions indicated by white squares are shown in the bottom panels. The right panels show merged images. Scale bars: 5 µm.

VPS45 is a member of SM family proteins, which are universal components for membrane fusion in eukaryotic cells. Distinct combinations of SM and SNARE (soluble N-ethylmaleimide-sensitive factor attachment receptor) proteins are thought to ensure specificity of membrane fusions [23]. In yeast, Vps45p binds to SNARE proteins Pep12p and Tlg2p and functions in trafficking to the vacuole [24]. Analogously, AtVPS45 forms a complex with TGN-localized SNARE proteins, which collectively play critical roles in vacuolar trafficking [22], [25]–[27]. In addition, recent studies have demonstrated crucial roles of VPS45 homologs in endocytic trafficking in yeast and animals [28]–[30]. As *ben2* mutant phenotypes suggest that AtVPS45 is involved in endocytic trafficking, we tested if AtVPS45 resides in endocytic route. Indeed, our colocalization study revealed that endocytosed FM4-64 rapidly colocalized with VPS45-GFP in root epidermal cells (Figure 3A). Consistently, VPS45-GFP or VPS45-RFP signals colocalized well with a TGN/EE marker VHA-a1-GFP and BEN1, but only marginally with a marker for Golgi apparatus SYP32-YFP (Figure 3B, Figure S3A). When we tested if PIN2-GFP proteins are detectable in BEN1 or BEN2-positive endosomes, we detected only a few colocalization under drug-untreated condition, probably because intracellular trafficking through the TGN/EE is a transient event (Figure S3B, S3C). However, upon treatment with BFA, both BEN1 and PIN2-GFP accumulated in BFA compartments (Figure S3B). On the other hand, VPS45-RFP was mainly detected at the periphery of agglomerated PIN2-GFP signals (Figure S3C).

Considering the proposed role of VPS45 in membrane fusion at the TGN [22] and the current model of early endocytic trafficking in *Arabidopsis* [31], it is reasonable to think that VPS45 resides in early endocytic route. The early endocytic trafficking defect observed in *ben2* mutant (Figure 1) is also consistent with the early endosomal localization of VPS45.

### *ben2* mutation influences BEN2/VPS45 localization

In order to know how the amino acid substitution by the *ben2* mutation affects the gene product, we generated a *VPS45-GFP* construct with the *ben2* mutant sequence (*VPS45D129N-GFP*) under the native promoter. Although transgenic plants expressing VPS45D129N-GFP fusion proteins exhibited punctate GFP signals in root epidermal cells, the GFP signals did not overlap with FM4-64 at early time point (Figure 3A, lower left panel), VPS45-RFP (Figure S3D), and BEN1 (Figure 3B, lower panel). In the presence of BFA, whereas VPS45-GFP partially colocalized with BEN1 mainly at the periphery of the BFA compartment, VPS45D129N-GFP did not show clear colocalization with BEN1 (Figure 3B, right panels). Thus, it seems that the *ben2* mutation compromised VPS45 function by affecting its association with TGN/EE. To examine the effect of *ben2* mutation on the subcellular localization of VPS45 protein in more detail, we tested prolonged incubation with the endocytic tracer FM4-64, which is known to stain late endocytic compartments and vacuolar membrane in time-dependent manner [32]. As shown in Figure 3A, after prolonged incubation, whereas most of the VPS45-GFP positive endosomes were labeled with FM4-64, some other intracellular FM4-64 signals were not colocalized with VPS45-GFP in root epidermal cells, suggesting that late endocytic compartments were also labeled at these time points (Figure 3A). In contrast, in VPS45D129N-GFP expressing seedling roots, most of the GFP-positive endosomes were not clearly labeled with FM4-64 over two hours. We also used an acidotropic probe LysoTracker red to visualize endosomal compartments. Whereas signals from VPS45-GFP and LysoTracker partially overlapped, VPS45D129N-GFP did not show major overlap (Figure S3D). Immunostaining with anti-SEC21 (a Golgi marker) and anti-BiP (an ER marker) also revealed no colocalization with VPS45D129N-GFP. Together, these results suggest that the *ben2* mutation caused mislocalization of VPS45D129N-GFP protein to a cryptic compartment other than ER, Golgi apparatus, TGN/EE, and the late compartments.

To gain more insight into the mechanistic impact of the *ben2* (D129N) mutation on the function of VPS45 protein, we examined whether the interaction between VPS45-GFP and TGN-localized Qa-SNARE SYP4 is affected by the D129N mutation. For this purpose, we immunoprecipitated VPS45-GFP and VPS45D129N-GFP with an anti-GFP antibody. As shown in Figure S3E, SYP4 was co-immunoprecipitated with VPS45-GFP, consistent with previous reports [22], [25]. However, SYP4 protein did not co-immunoprecipitate with VPS45D129N-GFP even though SYP4 protein was detectable in total protein extract, suggesting that the *ben2* mutation has compromised the physical association between VPS45 and SYP4.

### BEN1- and BEN2-dependent trafficking is involved in polar localization of PIN proteins

**Figure 4:**
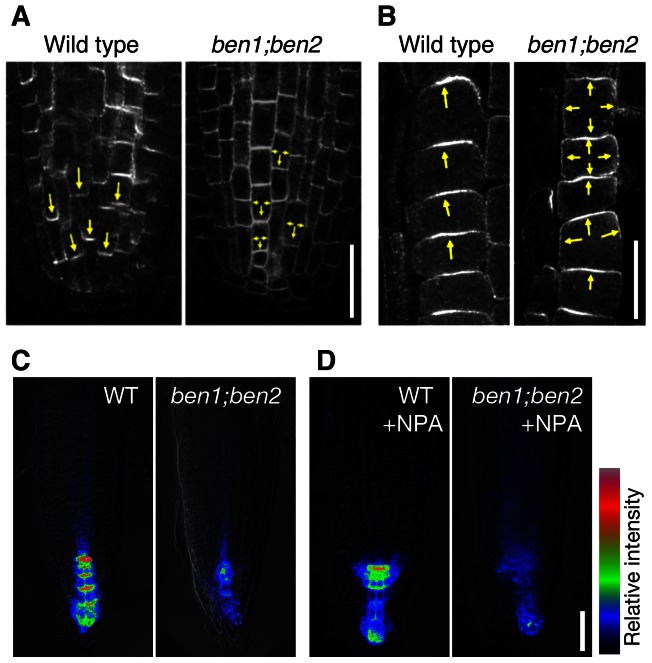
*BEN1* and *BEN2* genes are required for polar localization of PIN proteins. (A,B) Antibody staining of endogenous PIN1 protein in vascular tissue (A) and PIN2 on epidermal cells (B) of wild type and *ben1; ben2* double mutant roots. Arrows indicate polar localization of PIN proteins. (C,D) *ben* mutations and chemical inhibitor of auxin transport collectively diminish auxin response maxima. Auxin response maxima were visualized by DR5rev::GFP with color coding of signal intensities. Seedlings of indicated genotypes (3d) were transferred to mock (C) or NPA-containing media (50 µM) and grown for 2 days before imaging. Scale bars: 20 µm for (A,B); 50 µm in (D) for (C,D).

In order to test the biological relevance of BEN1- and BEN2-dependent trafficking through the TGN/EE, we examined the subcellular localization of PIN proteins in wild type and mutant roots. Our immunofluorescent study and quantitative analysis revealed that *ben1* and *ben2* mutation compromised the polar localization of PIN1 in the root tip (Figure 4A, Figure S4A). Whereas polar localization of PIN2 protein was not significantly affected in *ben1* and in *ben2* single mutants, the polar localization was significantly impaired in *ben1; ben2* double mutant. And in severe cases, PIN2 signals were almost non-polar in the root epidermal cells (Figure 4B, Figure S4B).

For polarization of PIN proteins, a tissue-dependent feedback regulation by auxin has been demonstrated [33]. A well-characterized example is root endodermal cells, where auxin induces PIN1 localization to the inner lateral side [33]. As previously described, treatment with naphthalene-1-acetic acid (NAA) induced significant enrichment of PIN1 to the inner side in wild type roots (Figure S4C, S4D). However, in the *ben1* and *ben2* mutants as well as *ben1; ben2* double mutant, PIN1 polarity is less pronounced without exogenously applied auxin and treatment with auxin did not dramatically induce relocation of PIN1 proteins (Figure S4C, S4D). As TGN/EE could be a sorting platform for trafficking pathways including secretion and vacuolar targeting [31], [34], we speculated that defects in BEN1 and BEN2 might affect multiple trafficking pathways. We expected that if secretion is severely impaired in *ben1* or *ben2* mutant, we might be able to see defect in recovery of PM proteins by a fluorescence recovery after photobleaching (FRAP) analysis. To test this possibility, we performed a FRAP experiment using PIN2-GFP. As shown in Figure S5A, when PIN2-GFP in a root epidermal cell was photobleached, the PM signal was recovered over 4 hours to approximately 13% of the initial signal intensity in *PIN2-GFP; eir1* line as control. Similarly, *ben1* and *ben2* mutants also exhibited recovery of the PM signals, approximately to 15 to 16% on average, revealing no discernible defect in the recovery rate (Figure S5B). Next, we evaluated if vacuolar targeting of PIN2 is affected. For this purpose, we incubated seedlings in the darkness before observation, to allow accumulation of GFP in the lytic vacuole [35], [36]. As reported previously, this allowed detection of vacuolar localized GFP signals in PIN2-GFP expressing root epidermal cells [36] (Figure S5C, S5D). Interestingly, *ben1* and *ben2* mutants as well as *ben1; ben2* double mutants tended to have weaker vacuolar GFP signals than control line did (Figure S5C, S5D), implying possible involvement of BEN1 and BEN2 also in vacuolar transport.

Taken together, these results support a scenario in which BEN1 and BEN2 are involved in intracellular trafficking including early endosomal trafficking through the TGN/EE that is required for polar localization of PIN1 and PIN2 proteins.

### BEN1- and BEN2-dependent trafficking is required for root architecture

Next, we addressed if BEN1- and BEN2-dependent trafficking is required for auxin-dependent developmental processes. As described above, whereas *ben1* and *ben2* single mutants exhibited only mild defects in terms of root growth, *ben1; ben2* double mutant showed a stronger defect (Figure S1D, S1E). Thus, our finding is consistent with previously suggested role of VPS45 in supporting plant growth [25], although *ben2* single mutant exhibited only minor phenotypes probably due to the hypomorphic nature of the *ben2* mutant allele. To gain more insights into the developmental roles of BEN1/MIN7 and BEN2/VPS45, we characterized the phenotypes in terms of localization of PIN proteins and auxin response gradient, mainly focusing on *ben1; ben2* double mutant.

Consistent with the PIN polarity defects as well as root growth defects, auxin response maxima as visualized by DR5rev::GFP [11] was reduced in *ben1; ben2* double mutant roots (Figure 4C) and it was further decreased in the presence of a polar auxin transport inhibitor naphtylphthalamic acid (NPA) (Figure 4D). Whereas the growth of primary roots was significantly slower, the formation of lateral roots was not inhibited in *ben1; ben2* plantlets, causing a short and bushy appearance of root system architecture (Figure S6A).

**Figure 5:**
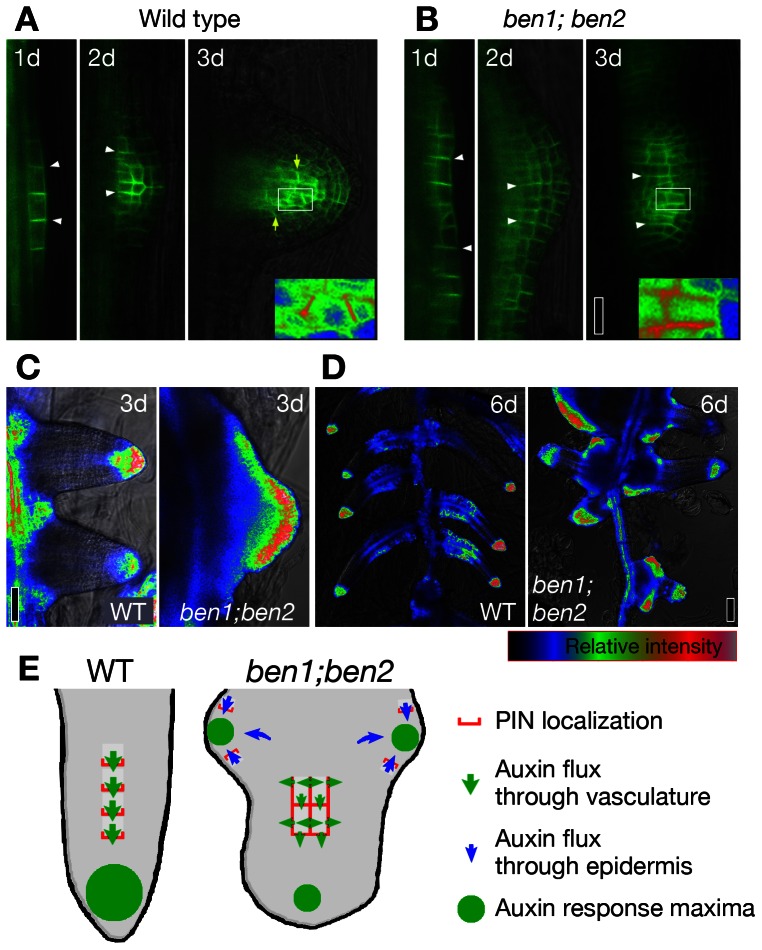
*ben* mutations alter the pattern of organ initiation and primordia morphology. (A,B) Localization of PIN1-GFP in developing LRPs. PIN1-GFP localizes to the anticlinal sides of young LRPs (white arrowheads) and gradually shifts its localization toward the tip of developing LRP within three days (A; yellow arrows). Relocation was less clear in the *ben1; ben2* double mutant (B). (C,D) Auxin response maxima as visualized by DR5rev::GFP reporter. Whereas sharp peaks were formed at the tips of LRPs in wild type, DR5 expression was broader in malformed LRPs of *ben1; ben2* three days after the onset of induction (C). Sharp peaks of auxin response maxima were maintained over time in wild type. In contrast, new auxin response maxima were generated in the base of LRPs in *ben1; ben2* (D). (E) A model to explain altered root architecture. PIN1 relocates towards the tip of LRP and facilitates directional auxin flow in provascular tissue (green arrows), resulting in generation of auxin maxima at the tip of LRP (green circle). PIN relocation is compromised in *ben1; ben2* double mutant. PIN1 is ectopically expressed in the epidermis by unknown mechanism, which in turn generates atypical auxin flow (blue arrows). Scale bars: 20 µm in (B) for (A,B); 50 µm in (C); 100 µm in (D).

To investigate the roles of *BEN* genes in lateral root (LR) development, we induced LR formation by auxin and followed the development of primordia. When grown in the presence of NAA, formation of lateral root primordia (LRP) is induced in wild type seedlings within 3 days with gradual relocation of PIN1-GFP from anticlinal sides to tipward of newly formed LRP [10] (Figure 5A). In *ben1; ben2* double mutant, PIN1-GFP did not efficiently relocate (Figure 5B), LRPs were deformed and auxin response maxima less confined to root tips (Figure 5C). Later on, wild type LRPs continued growing, forming dense array of lateral roots with strong DR5 response maximum at each root tip (Figure 5D). At the same time point, however, additional primordia with DR5 maxima were formed on growing LRPs in *ben1; ben2* double mutant (Figure 5D). In such LRPs, PIN1-GFP was ectopically expressed in the epidermis of the mutant LRPs and polarized as if it might have created ectopic auxin maxima (Figure 5E, Figure S6B), although PIN1 in provascular tissues drives auxin flow toward the tips of developing root primordia in normal course as well as auxin-induced LRP initiation [10] (Figure 5E).

### BEN1 and BEN2 are required for multiple auxin-dependent developmental processes

Because dynamic relocation of PIN proteins is involved in multiple developmental processes including root and shoot organogenesis, embryonic patterning, and vasculature venation patterns [10], [11], [37], we next examined if BEN-dependent trafficking is commonly required for these processes. Whereas *ben1* and *ben2* single mutants showed only moderate shoot growth defect, *ben1; ben2* mutant exhibited severely impaired shoot growth (Figure S6C). We also noticed that positions of siliques were often irregular in the *ben1; ben2* double mutant plants (Figure S6D). During embryogenesis, whereas *ben1* and *ben2* single mutants exhibited only moderate patterning defect (Figure S6F), patterning in developing embryos were more often disrupted in the *ben1; ben2* mutant (Figure S6E, S6F). In such abnormal embryos, polar localization of PIN1-GFP and auxin response maxima were less pronounced (Figure S6G, S6H). Similarly, whereas *ben1* single mutant exhibited a moderate defects in cotyledon venation pattern as reported previously [19] and *ben2* single mutation did not show discernible defect, *ben1; ben2* double mutant exhibited a severe venation pattern defects (Figure S7A–S7C). During the course of leaf primordial development, PIN1 expression precedes the development of vasculature and PIN1 polarity typically points toward preexisting veins when the PIN1 expression domain becomes connected [37] (Figure S7D). However, PIN1-GFP expression often remained disconnected in *ben1; ben2* leaf primordia with less pronounced polar localization of PIN1-GFP at the PM (Figure S7E).

## Discussion

The data presented here demonstrated that *BEN1* and *BEN2* genes have clear biological relevance in polar PIN localization, local auxin response, patterning and plant architecture. PIN-dependent regulation of local auxin distribution is a plant-specific mechanism, which is repeatedly utilized in different aspects of growth and development [2]. Polar localization of PIN proteins requires clearance from the PM by endocytosis [38], [39], retargeting to and retention at the polar domain [18], [40]. This work specifically reveals that the trafficking at the level of early endosomes is a critical mechanism for polar distribution of proteins exemplified by PIN auxin transporters.

In the current study, we showed that *BEN2* is identical to *AtVPS45*, which has been known to function in the vacuolar targeting pathway. Reportedly, when the VPS45 function is inactivated by RNAi, vacuolar cargo proteins containing the C-terminal vacuolar sorting determinants are mistargeted and instead transported to the apoplast [25]. Our finding may represent a parallel situation, as the *ben2* mutation compromised vacuolar localization of PIN2-GFP and together with *ben1* mutation caused a relatively strong PIN2 polarity defect at the PM. It is also possible that reduced activities of BEN1/MIN7 and BEN2/VPS45 might have caused dysfunction of TGN/EE to compromise multiple trafficking pathways that include vacuolar targeting and endocytic recycling. Results of our ultrastructural analysis fit with the later hypothesis, because *ben2* mutation, which might interfere with membrane fusion at the TGN/EE, resulted in accumulation of enlarged vesicle-like structures especially when combined with *ben1* mutation, as if membrane budding might be inhibited and/or identity of the accumulating vesicles might be altered. Although we still do not know the detailed mechanisms regulating PIN trafficking at the TGN/EE, our results suggest that BEN1/MIN7 and BEN2/VPS45 are important components.

In addition to the steady-state polar localization of PIN proteins in established tissues, dynamic changes of PIN localization are involved in plant development. It has also been demonstrated that the polar localization and amount of PIN proteins at the PM are controlled by different environmental stimuli [13], [14] as well as by endogenous cues [10], [11], [37]. Plant hormones auxin and cytokinin have strong impact in regulating PIN level and/or localization. Interestingly, each of these plant hormones regulates PINs by different modes (i.e. transcriptional and posttranslational control). For example, whereas auxin inhibits endocytosis via regulation of an auxin receptor ABP1 [41]–[43], it also stimulates relocation of PIN1 by a manner that requires transcriptional regulation, probably through another class of auxin receptor TIR1/AFBs [33]. On the other hand, cytokinin not only modifies transcription of PIN genes in root meristem [44]–[47] but also promotes degradation of PIN1 protein in LRP [48]. A possible link between cytokinin-regulated PIN degradation and *BEN* genes has also recently been provided [48], although the underlying mechanisms remain elusive. Here, we showed that defects in early endosomal components had strong impact on auxin-mediated PIN relocation as well as developmental processes that involve dynamic changes of PIN polarity. We speculate that efficient trafficking through TGN/EE is involved in rapid changes of PIN polarity and/or levels in response to the stimuli. The mechanism by which multiple stimuli regulate PIN localization via BEN-dependent trafficking is an important issue to be addressed in future.

Although our results highlight the roles of early endosomal trafficking in PIN-dependent regulation of plant development, many other proteins might be transported via BEN1- and BEN2-dependent pathways. In this respect, it is noteworthy that roles of trafficking at the level of TGN/EE on defense responses and vacuolar targeting are recently emerging [25], [27], [48], [49].

## Materials and Methods

### Plant materials and phenotypic analysis

Following *Arabidopsis thaliana* mutants and transgenic lines have been described: *PIN1-GFP* [10], *ben1-1*, *ben1-2*, *ben2-1* [19], *DR5rev::GFP* [11], *VHA-a1-GFP* [50], Wave 22Y [51]. PIN2-GFP [52] was introduced into *ben1-1*, *ben2* and *ben1-1; ben2* background by genetic crossing. Measurements of root length and clearing of seedlings and embryos were performed as described previously [19].

### Chemical treatments

Treatment with BFA (Invitrogen B7450) and staining with FM4-64 (Sigma S6689) were performed as described [19]. ES1 [20] (3.6 mM stock in DMSO) was diluted with liquid media. For LRP induction, young seedlings (3d) were transferred to solid plate containing 10 µM of NAA and vertically grown for indicated period. For evaluating auxin-induced PIN1 lateralization, seedlings were treated with 10 µM of NAA for 4 hours as described [33].

### Immunodetection and microscopy

Whole-mount immunolocalization on *Arabidopsis* roots were performed as described [19]. Antibodies were diluted as follows: rabbit anti-PIN1 (1∶1000) [41], rabbit anti-PIN2 (1∶1000) [53], rabbit anti-MIN7/BEN1 (1∶1000) [49], rabbit anti-SEC21 (1∶1000; Agrisera AS08 327), rabbit anti-BiP (1∶1000; Agrisera AS09 481), Cy3-conjugated secondary anti-rabbit (1∶600; Sigma C2306) antibodies. For staining with LysoTracker red, 1 mM stock in DMSO (Invitrogen L7528) was diluted with liquid media for *Arabidopsis* to make 2 µM solution and incubated for one hour before observation. Fluorescence imaging was done either by Carl Zeiss LSM5 exciter or LSM710 confocal microscopes. For calculating polarity index of PIN1 and PIN2, medial sections of root tips or paradermal sections through root epidermis were imaged at least from 10 roots in each line. Signal intensities of the polar domains and lateral PM were measured by ImageJ using the line function and statistic analyses were performed using PRISM software (version 5.0a, GraphPad Software, Inc.). Photobleaching was performed essentially as described [54] using at least 6 seedlings from each genotype. To visualize vacuolar localization of GFP in PIN2-GFP expressing lines, seedlings on vertical plates were kept in the darkness for 5 hours before observation. Vacuolar signals were evaluated by using color-coded images of paradermal confocal sections, typically containing 15 to 20 epidermal cells per root. Transmission electron microscopy was performed as described [19].

### Molecular cloning of *BEN2* gene and DNA construction

Polymorphic F_2_ seedlings obtained from crossing *ben2* mutant with a Landsberg *erecta* plant were treated with BFA and screened for *ben2* mutant phenotype by epifluorescense microscopy. DNA was isolated from each of 220 homozygous F_2_ plants and subjected to fine mapping. The *ben2* mutation was mapped between two markers F22K20-Eco72I (28.98 Mb) and T5M16-IPI (29.12 Mb) on chromosome 1. In this 140-kb region, we found a mutation in AtVPS45 gene (At1g77140). For genotyping, the *ben2-1* mutation was detected by PCR with primers BEN2-863D(m): 5′-GGTTTTTTATATTGCAGGAGTATTCTGC-3′ and BEN2-970R: 5′-CGACAACTGCGGGGATCA-3′ followed by digestion with restriction enzyme PstI. To generate VPS45-GFP and VPS45-RFP fusion constructs under control of CaMV 35S promoter, *VPS45* sequence was amplified from *Arabidopsis* cDNA (ecotype Col-0) with primers VPS45-GFP B1: 5′-GGGGACAAGTTTGTACAAAAAAGCAGGCTATGGTTTTGGTTACGTCTGTGCGT-3′ and VPS45-GFP B2 : 5′-GGGGACCACTTTGTACAAGAAAGCTGGGTCCACCATATGGCTACCTGATC-3′. The PCR-amplified VPS45 cDNA was cloned behind cauliflower mosaic virus 35S promoter (*P35S*) of Gateway-compatible binary vectors pH7FWG2 [55] and pK7RWG2 (https://gateway.psb.ugent.be/; *RFP* was kindly provided by Dr. Tsien, HHMI, UCSD). The resultant constructs pH7FWG2-*p35S::VPS45-GFP* and pK7RWG2-*p35S::VPS45-RFP* were transformed by agrobacterium-mediated floral-dip transformation procedure into *ben2-1* mutant and wild type Col-0, respectively. For generating genomic *VPS45* fused to *GFP* under control of native promoter, genomic fragment was amplified from wild type and *ben2* mutant with following primers: pVPS45-B1: GGGGACAAGTTTGTACAAAAAAGCAGGCTCGCAAAACGGTGCGTATTAGGAAAAT; VPS45-GFP-B2: GGGGACCACTTTGTACAAGAAAGCTGGGT T CACCATATGGCTACCTGATC. Amplified fragments were cloned into a binary vector pH7FWG,0 (https://gateway.psb.ugent.be/) to generate binary constructs pH7FWG-*pVPS45::VPS45-GFP* and pH7FWG-*pVPS45::VPS45D129N-GFP*, respectively. These constructs were transformed into Col-0 wild type plants. Transgenic plants were selected on solid media containing Kanamycin (25 mg/L) or Hygromycin (15 mg/L).

### Immunoprecipitation and immunoblot experiments

Immunoprecipitation from detergent extracts was carried out using the micro-MACS GFP-tagged protein isolation kit (Miltenyi Biotec), according to the manufacturer's instructions using 12 day-old *A. thaliana* plantlets (0.5 g) as the starting material. Immunoblot analysis was performed as described [27] with antibody against SYP4 (Uemura, T., unpublished) and anti-GFP antibody (Nacalai Tesque).

## Supporting Information

Figure S1 *ben1* and *ben2* mutations cause common phenotypes as well as distinct phenotypes, related to Figure 1. (A) PIN2 localization in BFA treated root epidermal cells of wild type, *ben1* and *ben2* single mutants and *ben1; ben2* double mutant. Typically agglomerated PIN2 signals were shown by arrowheads. (B) Vacuolar labeling by FM4-64 in wild type and *ben2* root epidermal cells after long incubation. (C) Quantitative evaluation of the ultrastructure of TGN/EE. Vesicle-like structures associated with the Golgi apparatus were quantified. Histogram shows frequency of vesicle-like structures with different sizes as indicated. (D) Synergistic effect of *ben1* and *ben2* mutations on seedling growth. (E) Quantification of root growth on solid plates containing BFA at different concentrations. Error bars indicate standard error (SE). Scale bars: 20 µm for (A); 4 µm for magnified images in (B).
(TIF)

Figure S2 Mapping and molecular cloning of *BEN2* gene, related to Figure 2. (A) Map-based cloning of *BEN2* locus. Numbers of recombination relative to the physical position on the chromosome 1 are indicated. (B) Exon-intron structure of AtVPS45 gene showing the site of nucleotide substitution in *ben2* mutant. (C) Complementation of morphological defect of *ben1; ben2* plantlet by VPS45-GFP. Scale bar: 1 cm.
(TIF)

Figure S3 Colocalization study using VPS45-XFP and subcellular markers, related to Figure 3. (A) Live imaging of VPS45-RFP (red) and subcellular markers (green) in root epidermal cells. VPS45-RFP signals largely overlapped with early endosomal marker VHA-a1-GFP (left panel). In contrast, VPS45-RFP did not colocalize with Golgi marker SYP32-YFP (Wave 22Y) (right panel). (B) Immunostaining of BEN1 (red) and PIN2-GFP (green). Whereas BEN1 partially colocalized with PIN2-GFP in the untreated epidermal cells (left), both PIN2-GFP and BEN1 accumulated to the center of the BFA compartment in BFA-treated cells (right). (C) PIN2-GFP and VPS45-RFP responded differently to BFA. The majority of VPS45-RFP localized to the periphery of the BFA compartment (right). (D) Colocalization between VPS45-GFP and organelle markers. Whereas VPS45-GFP colocalized very well with VPS45-RFP as control, VPS45D129N-GFP signals did not overlap with VPS45-RFP (left). Whereas VPS45-GFP marginally colocalized with LysoTracker and SEC21 signals, VPS45D129N-GFP did not colocalize with Lyso tracker, SEC21 (Golgi marker) and BiP (ER marker). Magnified views of the regions indicated by white squares are indicated in the bottom panels. The right panels show merged images. (E) Interaction between VPS45-GFP and Qa-SNARE SYP4 was affected by the *ben2* mutation. VPS45-GFP and VPS45D129N-GFP were extracted from plantlets and immunoprecipitated with anti-GFP antibody (top panel). Whereas Qa-SNARE SYP4 co-immunoprecipitated with wild type version of VPS45-GFP, co-immunoprecipitation of SYP4 with VPS45D129N-GFP was not detectable (bottom panel). Arrowheads indicate expected sizes of VPS45-GFP (∼92 kDa) and SYP4 (∼36 kDa). Scale bars: 5 µm in (A–D).
(TIF)

Figure S4 *ben* mutations reduce polar localization of PIN proteins, related to Figure 4. (A) Evaluation of asymmetric PIN1 localization in root stele cells of different genetic background. Polarity index concerning PIN1 localization in *ben1*, *ben2* and *ben1; ben2* double mutants were significantly reduced compared with that of wild type. Asterisks indicate statistic significance as compared with wild type (P<0.0001 by Mann-Whitney, two-tailed, non-parametric test). (B) Quantification of polar localization of PIN2 in root epidermal cells. Whereas *ben1* and *ben2* single mutations had only minor effect on PIN2 polarity, PIN2 localization was significantly affected in *ben1; ben2* double mutant background (asterisk, P<0.0001 by Mann-Whitney, two-tailed, non-parametric test). (C) Auxin-dependent changes of PIN1 polarity in endodermis cells. Upper panels and lower panels show PIN1 immunolocalization in mock-treated and NAA treated (10 µM for 4 h) roots, respectively. (D) Quantitative evaluation of PIN1 polar localization in endodermal cells. Graph shows ratio of lateral to basal signal intensity in endodermis cells with SE. Scale bars: 10 µm.
(TIF)

Figure S5 Characterization of trafficking defects in *ben* mutants, related to Figure 4. (A) FRAP analysis of PIN2-GFP in root epidermal cells. Images display the region of roots before photobleaching (Pre), just after photobleaching (Post), and fluorescence recovery at indicated time points. Arrowheads indicate recovered signals at the apical PM. (B) Quantification of recovery at the apical PM. The intensities of apical PM signals deceased to below 5% after photobleaching, but recovered to 13 to 16% on average in control line (*PIN2-GFP; eir1*) as well as in *ben1*, *ben2* and *ben1; ben2* mutants harboring PIN2-GFP. (C) Visualization of vacuolar GFP signals in seedling roots. Arrowheads in *PIN2-GFP; eir1* and *PIN2-GFP; ben1-1; eir1* lines indicate typical vacuolar GFP signals. (D) Frequency of seedlings in which vacuolar GFP signals were observed. 22 to 51 seedling roots from each genotype were evaluated as described in Materials and Methods. Scale bar: 20 µm in (C).
(TIF)

Figure S6 BEN1 and BEN2 are involved in organ formation and patterning, related to Figure 5. (A) Gross morphology of vertically grown wild type and *ben1; ben2* plantlets (10 d). Inset shows magnified view of 14 day-old *ben1; ben2* plantlet. (B) Elevated PIN1-GFP in the epidermis of developing LRP (arrowheads). Magnified view shows a color-coded image of the boxed region. (C) Shoot morphology of 6 week-old wild type and *ben* mutants. (D) Siliques are formed in irregular pattern in the *ben1; ben2* double mutant. (E) Patterning of embryonic root is severely disrupted in *ben1; ben2* double mutant. Arrowheads indicate lens-shaped cells typically found in wild type embryos. Abbreviations: g, globular stage; t, triangular stage; h, early heart stage. (F) Frequency of abnormal embryos at the globular- and post-globular stages. (G) Localization of PIN1-GFP in developing embryos. (H) Inspection of DR5::GFP expression in the developing embryos revealed less pronounced auxin response gradients in *ben1;ben2* embryos. Scale bars: 100 µm in (B); 1 cm for (D); 20 µm in (E); 10 µm in (G,H).
(TIF)

Figure S7 Vasculature venation pattern is affected by *ben1* and *ben2* mutations, related to Figure 5. (A) Pattern of venation in mature cotyledons of wild type and *ben* mutants. Arrowheads indicate unusual disconnected veins. (B) Schematic drawings of cotyledon venation pattern defects. Red dots represent defective sites. Frequency of venation defects. N indicates numbers of cotyledons examined. (D,E) Patterns of PIN1-GFP in the first pair of leaves. Whereas PIN1-GFP expressing veins tend to form loops from early stages of wild type leaf primordia (D), PIN1-GFP expressing regions often remained unconnected in *ben1; ben2* (arrowheads). Polar localization of PIN1-GFP was less pronounced in *ben1; ben2* leaf primordia. Scale bars: 0.5 mm for (A); 50 µm for (D,E).
(TIF)
