# Supplementary figures and images for: Cell Polarity and Patterning by PIN Trafficking through Early Endosomal Compartments in Arabidopsis thaliana
Source: PLoS Genet. 2013 May 30;9(5):e1003540. doi: 10.1371/journal.pgen.1003540 (PMC3667747; doi:10.1371/journal.pgen.1003540)

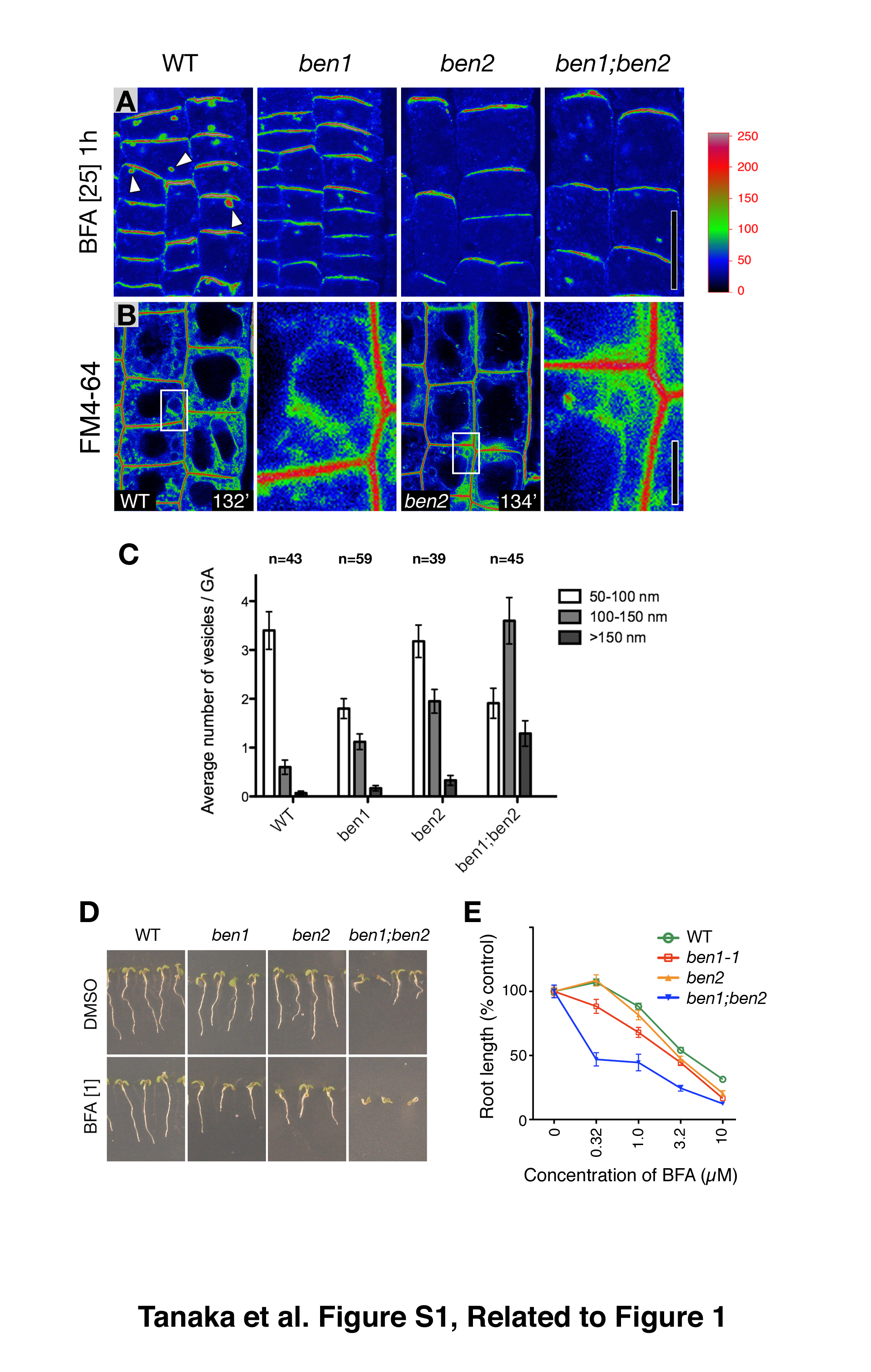

Supplement: Figure S1 — ben1 and ben2 mutations cause common phenotypes as well as distinct phenotypes, related to Figure 1. (A) PIN2 localization in BFA treated root epidermal cells of wild type, ben1 and ben2 single mutants and ben1; ben2 double mutant. Typically agglomerated PIN2 signals were shown by arrowheads. (B) Vacuolar labeling by FM4-64 in wild type and ben2 root epidermal cells after long incubation. (C) Quantitative evaluation of the ultrastructure of TGN/EE. Vesicle-like structures associated with the Golgi apparatus were quantified. Histogram shows frequency of vesicle-like structures with different sizes as indicated. (D) Synergistic effect of ben1 and ben2 mutations on seedling growth. (E) Quantification of root growth on solid plates containing BFA at different concentrations. Error bars indicate standard error (SE). Scale bars: 20 µm for (A); 4 µm for magnified images in (B). (TIF) [file pgen.1003540.s001.tif]

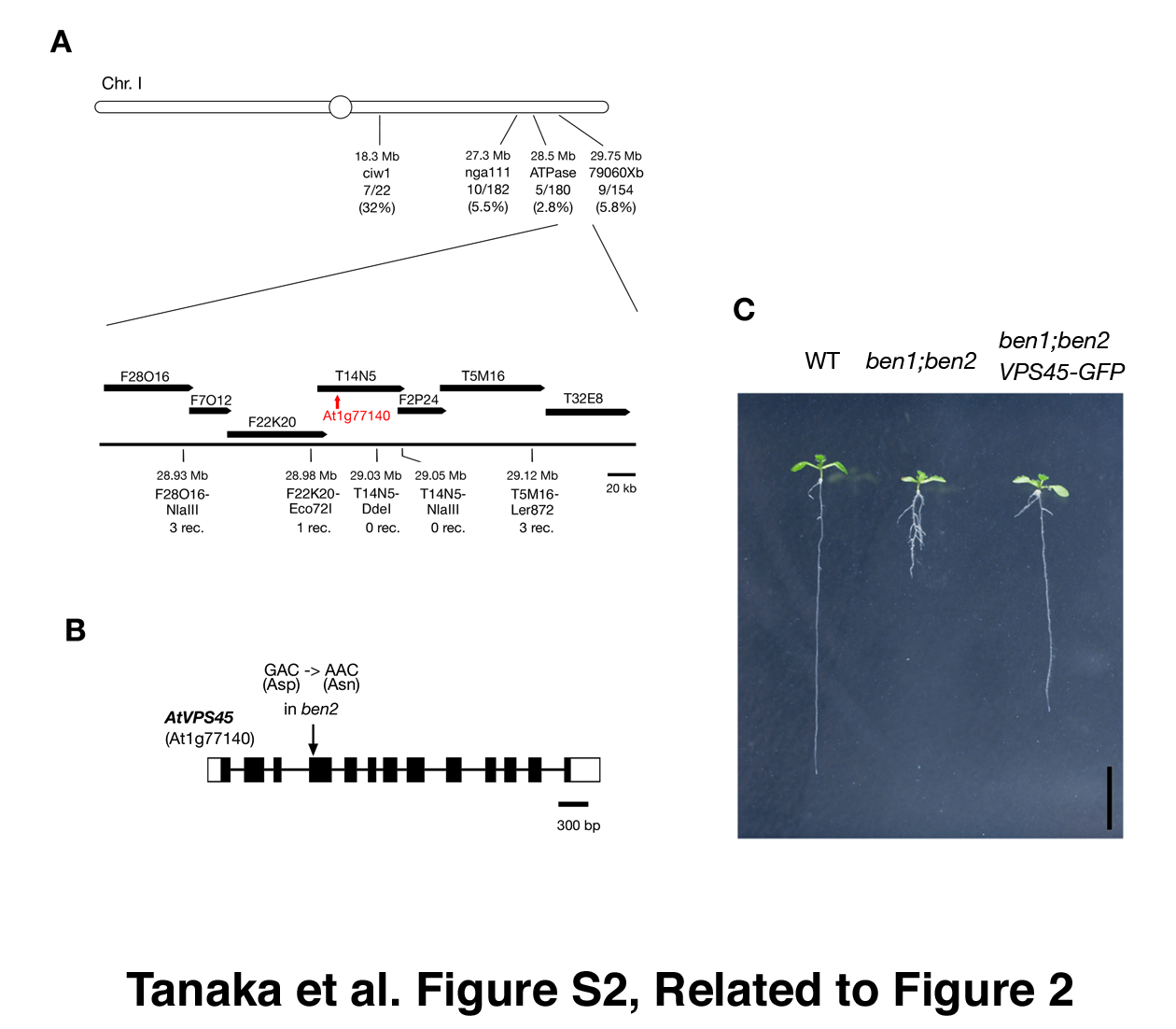

Supplement: Figure S2 — Mapping and molecular cloning of BEN2 gene, related to Figure 2. (A) Map-based cloning of BEN2 locus. Numbers of recombination relative to the physical position on the chromosome 1 are indicated. (B) Exon-intron structure of AtVPS45 gene showing the site of nucleotide substitution in ben2 mutant. (C) Complementation of morphological defect of ben1; ben2 plantlet by VPS45-GFP. Scale bar: 1 cm. (TIF) [file pgen.1003540.s002.tif]

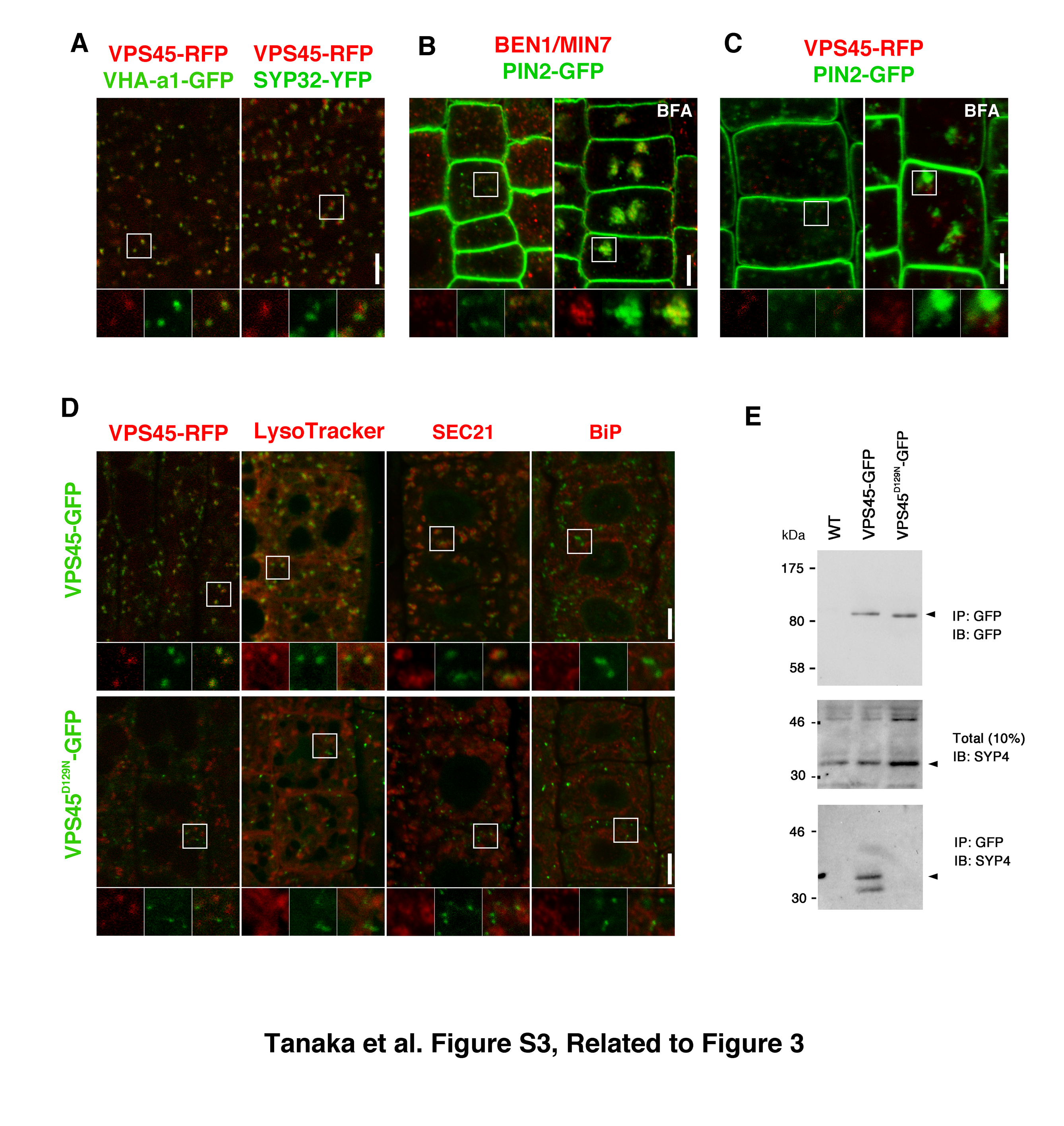

Supplement: Figure S3 — Colocalization study using VPS45-XFP and subcellular markers, related to Figure 3. (A) Live imaging of VPS45-RFP (red) and subcellular markers (green) in root epidermal cells. VPS45-RFP signals largely overlapped with early endosomal marker VHA-a1-GFP (left panel). In contrast, VPS45-RFP did not colocalize with Golgi marker SYP32-YFP (Wave 22Y) (right panel). (B) Immunostaining of BEN1 (red) and PIN2-GFP (green). Whereas BEN1 partially colocalized with PIN2-GFP in the untreated epidermal cells (left), both PIN2-GFP and BEN1 accumulated to the center of the BFA compartment in BFA-treated cells (right). (C) PIN2-GFP and VPS45-RFP responded differently to BFA. The majority of VPS45-RFP localized to the periphery of the BFA compartment (right). (D) Colocalization between VPS45-GFP and organelle markers. Whereas VPS45-GFP colocalized very well with VPS45-RFP as control, VPS45D129N-GFP signals did not overlap with VPS45-RFP (left). Whereas VPS45-GFP marginally colocalized with LysoTracker and SEC21 signals, VPS45D129N-GFP did not colocalize with Lyso tracker, SEC21 (Golgi marker) and BiP (ER marker). Magnified views of the regions indicated by white squares are indicated in the bottom panels. The right panels show merged images. (E) Interaction between VPS45-GFP and Qa-SNARE SYP4 was affected by the ben2 mutation. VPS45-GFP and VPS45D129N-GFP were extracted from plantlets and immunoprecipitated with anti-GFP antibody (top panel). Whereas Qa-SNARE SYP4 co-immunoprecipitated with wild type version of VPS45-GFP, co-immunoprecipitation of SYP4 with VPS45D129N-GFP was not detectable (bottom panel). Arrowheads indicate expected sizes of VPS45-GFP (∼92 kDa) and SYP4 (∼36 kDa). Scale bars: 5 µm in (A–D). (TIF) [file pgen.1003540.s003.tif]

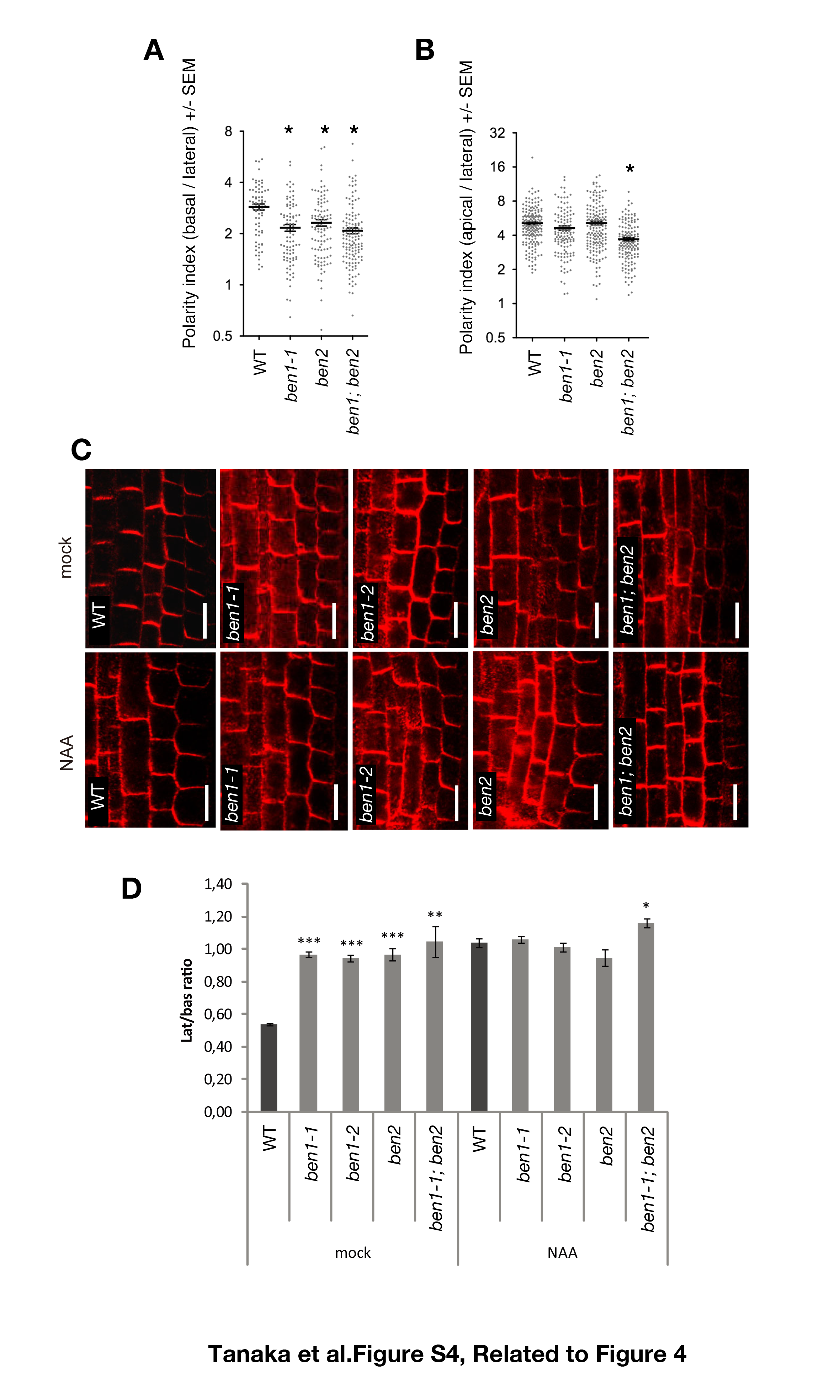

Supplement: Figure S4 — ben mutations reduce polar localization of PIN proteins, related to Figure 4. (A) Evaluation of asymmetric PIN1 localization in root stele cells of different genetic background. Polarity index concerning PIN1 localization in ben1, ben2 and ben1; ben2 double mutants were significantly reduced compared with that of wild type. Asterisks indicate statistic significance as compared with wild type (P<0.0001 by Mann-Whitney, two-tailed, non-parametric test). (B) Quantification of polar localization of PIN2 in root epidermal cells. Whereas ben1 and ben2 single mutations had only minor effect on PIN2 polarity, PIN2 localization was significantly affected in ben1; ben2 double mutant background (asterisk, P<0.0001 by Mann-Whitney, two-tailed, non-parametric test). (C) Auxin-dependent changes of PIN1 polarity in endodermis cells. Upper panels and lower panels show PIN1 immunolocalization in mock-treated and NAA treated (10 µM for 4 h) roots, respectively. (D) Quantitative evaluation of PIN1 polar localization in endodermal cells. Graph shows ratio of lateral to basal signal intensity in endodermis cells with SE. Scale bars: 10 µm. (TIF) [file pgen.1003540.s004.tif]

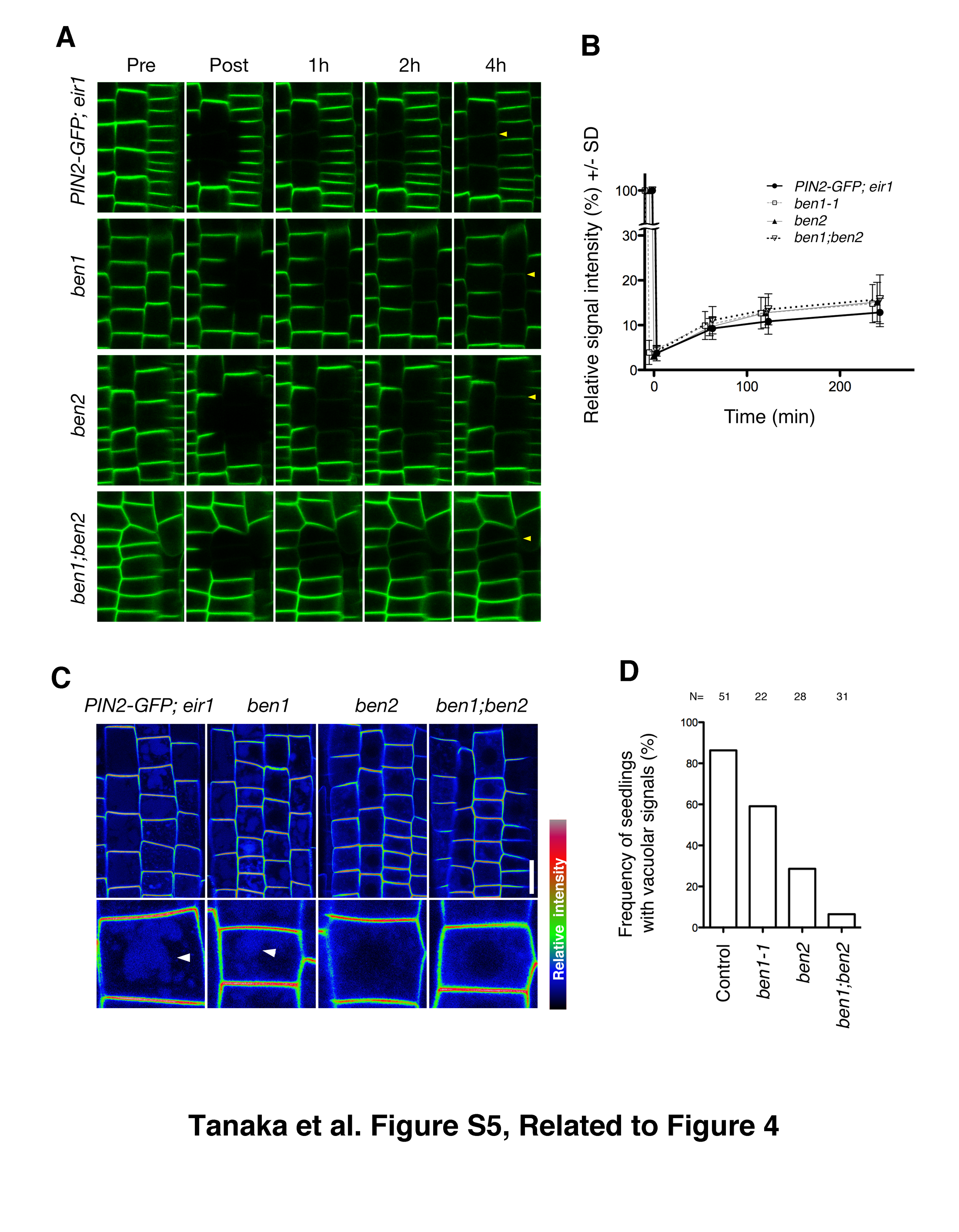

Supplement: Figure S5 — Characterization of trafficking defects in ben mutants, related to Figure 4. (A) FRAP analysis of PIN2-GFP in root epidermal cells. Images display the region of roots before photobleaching (Pre), just after photobleaching (Post), and fluorescence recovery at indicated time points. Arrowheads indicate recovered signals at the apical PM. (B) Quantification of recovery at the apical PM. The intensities of apical PM signals deceased to below 5% after photobleaching, but recovered to 13 to 16% on average in control line (PIN2-GFP; eir1) as well as in ben1, ben2 and ben1; ben2 mutants harboring PIN2-GFP. (C) Visualization of vacuolar GFP signals in seedling roots. Arrowheads in PIN2-GFP; eir1 and PIN2-GFP; ben1-1; eir1 lines indicate typical vacuolar GFP signals. (D) Frequency of seedlings in which vacuolar GFP signals were observed. 22 to 51 seedling roots from each genotype were evaluated as described in Materials and Methods. Scale bar: 20 µm in (C). (TIF) [file pgen.1003540.s005.tif]

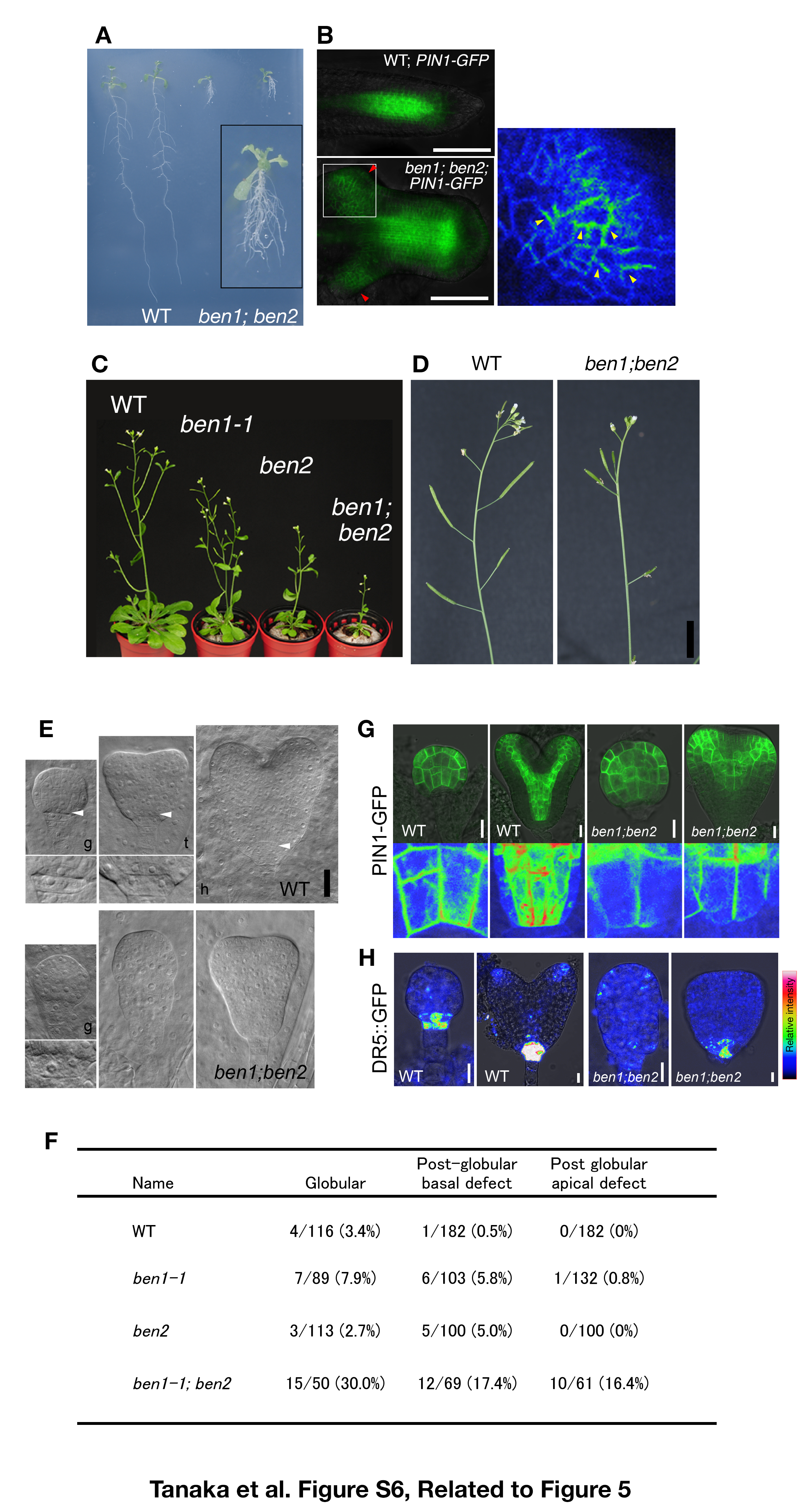

Supplement: Figure S6 — BEN1 and BEN2 are involved in organ formation and patterning, related to Figure 5. (A) Gross morphology of vertically grown wild type and ben1; ben2 plantlets (10 d). Inset shows magnified view of 14 day-old ben1; ben2 plantlet. (B) Elevated PIN1-GFP in the epidermis of developing LRP (arrowheads). Magnified view shows a color-coded image of the boxed region. (C) Shoot morphology of 6 week-old wild type and ben mutants. (D) Siliques are formed in irregular pattern in the ben1; ben2 double mutant. (E) Patterning of embryonic root is severely disrupted in ben1; ben2 double mutant. Arrowheads indicate lens-shaped cells typically found in wild type embryos. Abbreviations: g, globular stage; t, triangular stage; h, early heart stage. (F) Frequency of abnormal embryos at the globular- and post-globular stages. (G) Localization of PIN1-GFP in developing embryos. (H) Inspection of DR5::GFP expression in the developing embryos revealed less pronounced auxin response gradients in ben1;ben2 embryos. Scale bars: 100 µm in (B); 1 cm for (D); 20 µm in (E); 10 µm in (G,H). (TIF) [file pgen.1003540.s006.tif]

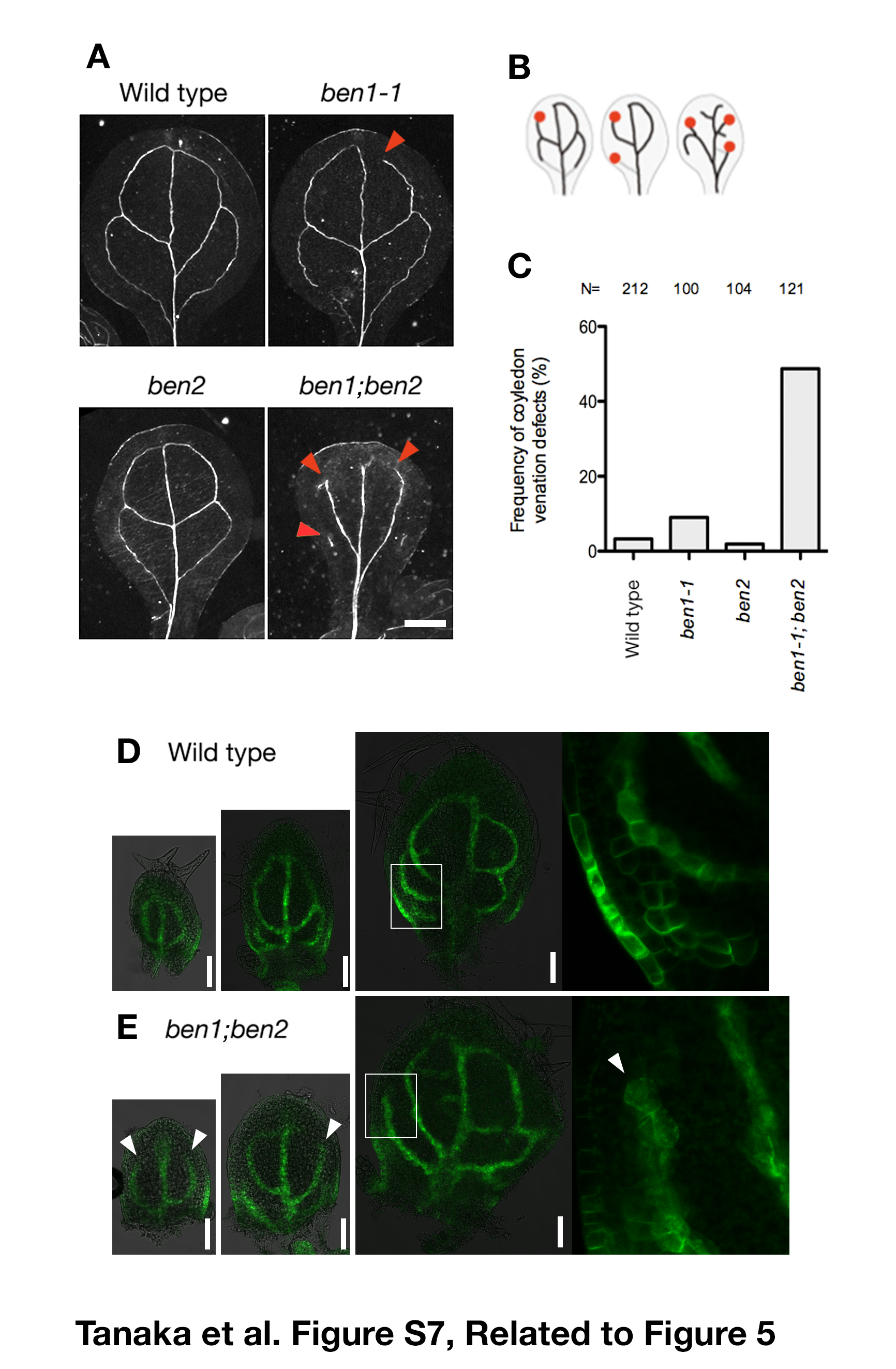

Supplement: Figure S7 — Vasculature venation pattern is affected by ben1 and ben2 mutations, related to Figure 5. (A) Pattern of venation in mature cotyledons of wild type and ben mutants. Arrowheads indicate unusual disconnected veins. (B) Schematic drawings of cotyledon venation pattern defects. Red dots represent defective sites. Frequency of venation defects. N indicates numbers of cotyledons examined. (D,E) Patterns of PIN1-GFP in the first pair of leaves. Whereas PIN1-GFP expressing veins tend to form loops from early stages of wild type leaf primordia (D), PIN1-GFP expressing regions often remained unconnected in ben1; ben2 (arrowheads). Polar localization of PIN1-GFP was less pronounced in ben1; ben2 leaf primordia. Scale bars: 0.5 mm for (A); 50 µm for (D,E). (TIF) [file pgen.1003540.s007.tif]
